# Dynamic evolution of the global burden of chronic urticaria: a comprehensive trend analysis from 1990 to 2021 and projections to 2050

**DOI:** 10.1097/JS9.0000000000003139

**Published:** 2025-08-07

**Authors:** Fang Cao, Zhining Tian, Hui Zhang, Qianying Yu, Jing Guo

**Affiliations:** Chengdu University of Traditional Chinese Medicine, Chengdu City, Sichuan, China

**Keywords:** epidemiological trends, global burden of disease, predictive analysis, public health, urticaria

## Abstract

**Background:**

Urticaria is a prevalent allergic skin condition that significantly affects patients’ quality of life and mental health. The incidence and prevalence of urticaria vary across regions and time due to globalization and environmental changes. Understanding the global prevalence and impact of urticaria is essential for developing effective prevention and treatment strategies.

**Objective:**

To assess the global burden of urticaria and project future trends, with a focus on its incidence, prevalence, and impact on public health.

**Methods:**

Utilizing data from the Global Burden of Disease 2021 database, this study assessed urticaria’s incidence, prevalence, mortality, and disability-adjusted life years from 1990 to 2021. Data were analyzed by gender, age, and sociodemographic index. Joinpoint analysis was employed to evaluate global and regional trend changes, while ARIMA and ES models projected the burden to 2050.

**Results:**

Between 1990 and 2021, global urticaria incident cases rose 37.9% (84.87 to 117.01 million) and prevalent cases 39.4% (47.95 to 66.84 million). Age-standardized rates remained nearly stable (incidence: 1529.24 to 1533.71/100 000; prevalence: 865.85 to 868.18/100 000; EAPC: 0.01% for both). Central and Eastern Europe had the highest burden. Children <5 years showed the highest incidence (3262.05/100 000). Burden was consistently higher in women. Projections to 2050 indicate a further ~25% increase in cases, driven primarily by demographics.

**Conclusions:**

Urticaria’s global burden is expected to rise, influenced by environmental changes, socioeconomic factors, and aging populations. Public health strategies should focus on environmental management, health education, resource optimization, and international cooperation to mitigate urticaria’s impact.

## Introduction

Urticaria is a common skin disease characterized by itchy wheals or angioedema, which can significantly impact patients’ quality of life and mental health^[1,2]^. Although urticaria is usually considered a mild skin problem, in people with chronic urticaria, symptoms can persist for months or even years, seriously affecting daily life^[3]^. While environmental factors have been hypothesized to contribute to urticaria burden based on mechanistic studies and regional observations, direct causal relationships between specific environmental changes and urticaria incidence patterns remain to be definitively established through large-scale epidemiological research. Some studies have suggested potential associations between air pollution, climate variability, and allergic disease prevalence, but robust evidence specifically linking these factors to urticaria burden at the population level is limited^[4]^. A comprehensive understanding of the global prevalence of urticaria and its impact on public health is critical to developing effective prevention and treatment strategies.

**HIGHLIGHTS**

- Urticaria affects quality of life and mental health, with its prevalence influenced by environmental changes, globalization, and socioeconomic factors, showing varied trends across regions.
- This study reveals an increasing global burden of urticaria, with projections indicating continued growth through 2050, especially in low-income regions and among high-risk populations, highlighting future challenges.
- The findings emphasize the need for targeted public health strategies, including environmental management, health education, and resource optimization to mitigate urticaria’s global impact.

At present, epidemiological studies on urticaria are mostly focused on specific countries or regions, and there are relatively few systematic analyses at the global level. The Global Burden of Disease (GBD) project provides detailed epidemiological data for the assessment of multiple diseases, including urticaria, covering key indicators such as incidence, prevalence, and disability-adjusted life years (DALYs)^[5,6]^. Previous research found that age-standardized incidence rates of urticaria continued to increase globally between 1990 and 2019, primarily due to environmental changes and socioeconomic factors^[7]^. However, detailed assessment of the latest global epidemiological trends, regional differences, and specific affected populations of urticaria is still insufficient, especially in the context of rapid changes in environmental factors and lifestyles in recent years^[8]^.

To address this knowledge gap, this study employs GBD 2021 data to deliver an updated and comprehensive assessment of urticaria incidence, prevalence, mortality, and DALYs at global, regional, and national levels from 1990 to 2021. The analysis is stratified by gender, age, and sociodemographic index (SDI) to identify the populations most affected by urticaria, thereby laying the groundwork for the development of targeted prevention and treatment strategies. Furthermore, we employed a time series analysis model to project the global burden of urticaria through 2050, aiming to evaluate potential trends in disease burden over the coming decades. This predictive analysis can aid in identifying high-risk groups and regions in the future, providing policymakers with forward-looking decision support and facilitating the development of more effective public health interventions. And our work has been reported in line with the TITAN criteria^[9]^.

## Methods

### Data acquisition and processing

This study collected global epidemiological data on urticaria from 1990 to 2021, utilizing the GBD database. The project represents a large-scale international effort aimed at estimating the health impacts of various diseases, injuries, and risk factors at global, regional, and national levels^[10]^. The dataset encompasses incidence, prevalence, mortality, and DALYs. To ensure consistency and comparability, all data were preprocessed and standardized in accordance with GBD standards. Data cleaning procedures included addressing missing values, managing outliers, and transforming data to meet the requirements of subsequent analyses^[11]^.

All data underwent rigorous quality control following GBD 2021 standards. Data preprocessing included systematic identification and handling of missing values using multiple imputation techniques, outlier detection using interquartile range methods, and standardization procedures ensuring comparability across countries with different diagnostic coding systems. Age-standardized rates were calculated using the GBD 2021 global age standard. Uncertainty intervals were calculated using 1000 bootstrap iterations.

### Descriptive analytics

By calculating the basic statistics of key indicators such as incidence, prevalence, mortality and DALYs provided by the GBD database, we reveal the distribution of disease burden by gender, age group, SDI region, GBD region, and country level. Graphs such as bar, line, and box plots were used to visualize the burden of disease by subgroup, with particular attention to the specific effects of age and sex on the burden of disease, and to assess differences between different regions and countries^[12]^. In addition, this study also delves into the social, economic, and environmental factors that may affect the burden of urticaria disease, providing a basis for subsequent trend analysis and predictive analysis.

### Trend analysis

In this study, we employed Joinpoint analysis to assess changes in the global disease burden of urticaria from 1990 to 2021^[13]^. Initially, we conducted a comprehensive subgroup analysis of urticaria incidence, prevalence, and disability-adjusted life years (DALYs) stratified by gender, age, sociodemographic index (SDI) region, Global Burden of Disease (GBD) region, and country level. We quantified the rate of increase or decrease in these indicators over time by calculating the estimated annual percentage change (EAPC) in age-standardized rates for each subgroup, where positive EAPC values indicate an increasing trend and negative values indicate a decreasing trend. The statistical significance of EAPC lies in its ability to provide a precise estimate of the rate of change in trends and to assess the significance of these trends through confidence intervals^[14]^. Additionally, we utilized cluster analysis to categorize GBD regions, with the aim of identifying regions exhibiting similar patterns of change in disease burden. This analysis enhances our understanding of the similarities and differences in trends across various regions, thereby offering a more accurate framework for inter-regional comparisons and potential public health strategies. By employing this methodology, we can find areas with similar disease patterns at the same SDI level or region, helping to spot shared causes and create targeted solutions.

### Prediction and analysis for 2030–2050

This study used two time series prediction models, including autoregressive integrated moving average (ARIMA) and exponential smoothing (ES) models, to predict the global disease burden trend of urticaria from 2030 to 2050. The ARIMA model is suitable for processing time series data with seasonal characteristics and non-stationarity^[15]^. ARIMA models were fitted using the auto.arima function in R software (version 4.3.0). The optimal model parameters were determined through systematic evaluation of information criteria, with final models showing AIC values of 156.32 for incidence data and 163.45 for prevalence data, and corresponding BIC values of 168.47 and 175.89, respectively. The selected models followed ARIMA^[1,1]^ specification for incidence trends and ARIMA^[1,2]^ for prevalence trends. Stationarity was confirmed using the Augmented Dickey-Fuller test (*P* < 0.001), and model diagnostics included Ljung-Box tests for residual autocorrelation (all *P* > 0.05) and Shapiro-Wilk tests for residual normality (*P* > 0.05). ES models utilized the ETS framework with additive error and trend components. Model selection based on AIC showed values of 148.6 for incidence and 151.3 for prevalence. Smoothing parameters were optimized with alpha parameters of 0.85 and 0.78, and beta parameters of 0.23 and 0.31 for incidence and prevalence, respectively^[16]^. We first performed stationarity testing and necessary difference processing on the time series data, and then used cross-validation methods (walk-forward validation approach) to evaluate the prediction performance of the model to ensure the accuracy of model selection and the reliability of the prediction results. We further assessed the accuracy and confidence of the forecasts by calculating uncertainty intervals for the forecast values.

## Results

### Trends in the global burden of urticaria

**Figure 1.**
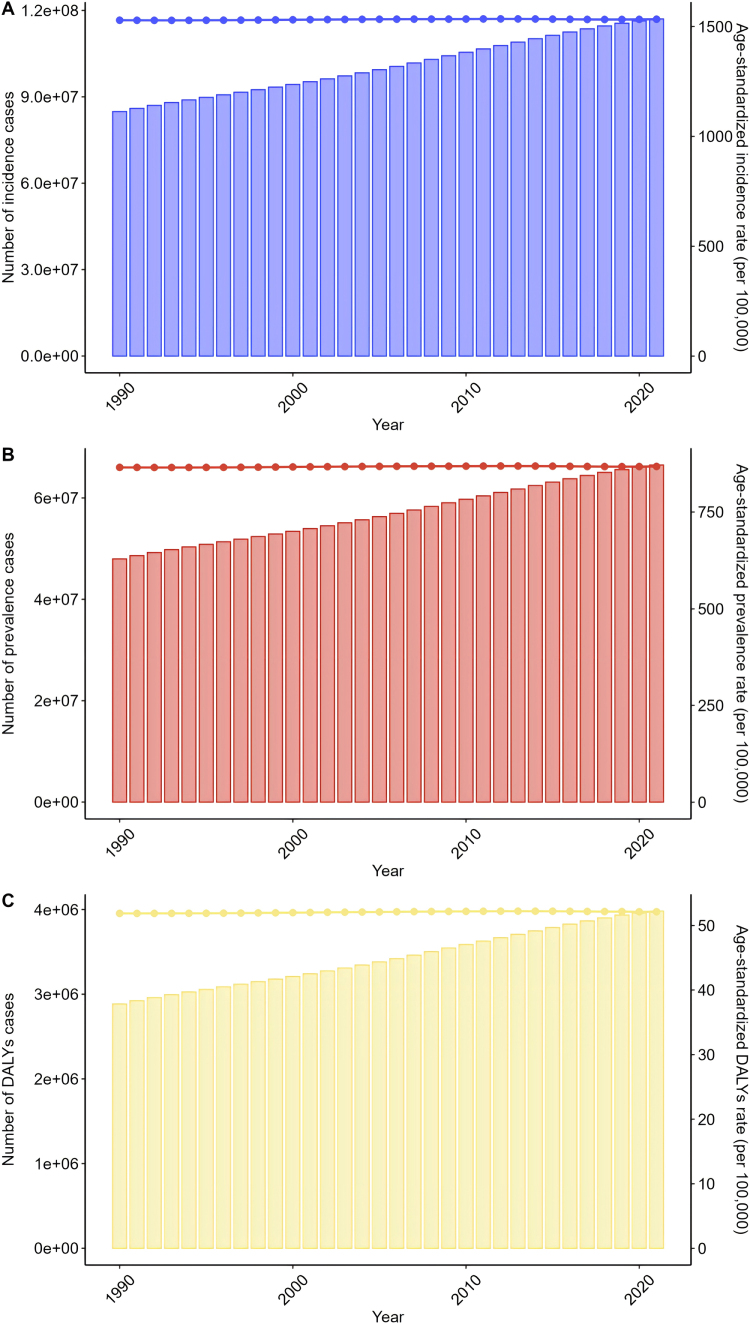
Global trends in urticaria from 1990 to 2021.

From 1990 to 2021, global prevalence and incidence of urticaria have risen. In 2021, the number of incident cases reached 117 014 587 (95% UI: 104 106 688–131 017 656), a significant increase from 84 870 288 in 1990 (95% UI: 74 471 430–96 206 256). The global age-standardized incidence rate in 2021 was 1533.71 per 100 000 people (95% UI: 1358.35–1726.07), slightly up from 1529.24 per 100 000 in 1990 (95% UI: 1355.95–1720.36), with an EAPC of 0.01 (95% CI: 0.01–0.02) (Fig. 1, Supplementary Digital Content Table 1, available at: http://links.lww.com/JS9/E869). Although the global age-standardized incidence and prevalence rates showed statistically significant increases from 1990 to 2021, the magnitude of change was modest, with estimated annual percentage change (EAPC) values of approximately 0.01% annually. While statistically significant due to large sample sizes, the clinical and public health significance of such small annual changes requires careful interpretation. The minimal increase suggests that urticaria burden has remained relatively stable at the global level, with population growth primarily driving absolute increases in case numbers rather than fundamental changes in disease incidence rates.

In 2021, the prevalence of urticaria cases was 66 843 473 (95% UI: 59 240 738–74 912 351), up from 47 949 600 in 1990 (95% UI: 42 276 752–54 859 774). The age-standardized prevalence rate in 2021 was 868.18 per 100 000 people (95% UI: 770.06–983.86), with minimal change from 865.85 per 100 000 in 1990 (95% UI: 761.81–980.76), and an EAPC of 0.01 (95% CI: 0.01–0.02) (Fig. 1, Supplementary Digital Content Table 2, available at: http://links.lww.com/JS9/E870).

Regarding disability-adjusted life years (DALYs), the global DALYs for urticaria in 2021 were 3 980 786 years (95% UI: 2 615 626–5 658 917), a significant increase from 2 883 966 years in 1990 (95% UI: 1 901 101–4 148 607). The age-standardized DALYs rate in 2021 was 52.11 years per 100 000 people (95% UI: 34.21–74.63), slightly up from 51.86 years per 100 000 people in 1990 (95% UI: 34.25–74), with an EAPC of 0.02 (95% CI: 0.02–0.03) (Fig. 1, Supplementary Digital Content Table 3, available at: http://links.lww.com/JS9/E871).

### Regional differences

Significant regional differences exist in the incidence, prevalence, and DALYs of urticaria. The highest incidence and prevalence rates were observed in Central and Eastern Europe, while Western Europe and Oceania had relatively lower rates. From 1990 to 2021, South Asia showed a significant upward trend in incidence, with an EAPC of 0.03 (95% UI: 0.03–0.04). Conversely, high-income Asia Pacific experienced a downward trend, with an EAPC of −0.02 (95% UI: −0.02–0.02).

In terms of DALYs, regions such as Central Europe, Eastern Europe, and Central Asia exhibited significantly higher DALYs rates, while East Asia and Oceania showed lower DALYs. South Asia exhibited a significant increase in DALYs, while high-income Asia Pacific experienced a marked decline.

**Figure 2.**
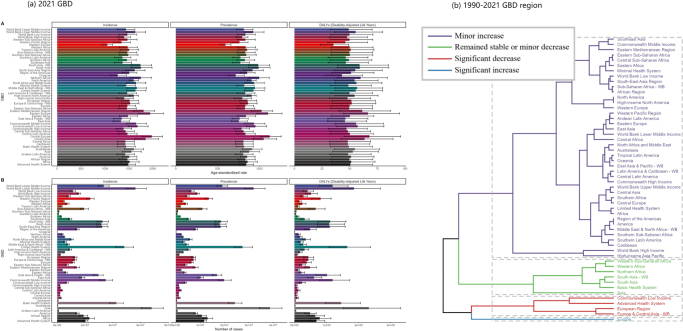
Age-standardized incidence, prevalence, and DALYs from the 2021 GBD by region and trends from 1990 to 2021.

Globally, there are significant regional differences in the incidence, prevalence, and DALYs of urticaria. Specifically, Central Europe and Eastern Europe have the highest incidence and prevalence rates, while Western Europe and Oceania have relatively lower rates. These regional differences are also reflected in the distribution of DALYs, with Central Europe, Eastern Europe, and Central Asia showing significantly higher DALYs rates compared to other regions, while East Asia and Oceania have lower DALYs rates (Fig. 2a). From 1990 to 2021, South Asia exhibited a significant upward trend in incidence, with an EAPC of 0.03 (95% UI: 0.03–0.04), whereas the incidence in High-income Asia Pacific showed a significant downward trend, with an EAPC of −0.02 (95% UI: −0.02–0.02). A similar trend is observed in prevalence rates, with significant growth in South Asia and a marked decline in High-income Asia Pacific. Regarding changes in DALYs, South Asia showed a significant increase, while High-income Asia Pacific experienced a notable decrease (Fig. 2b).

### Differences in the different SDI levels

**Figure 3.**
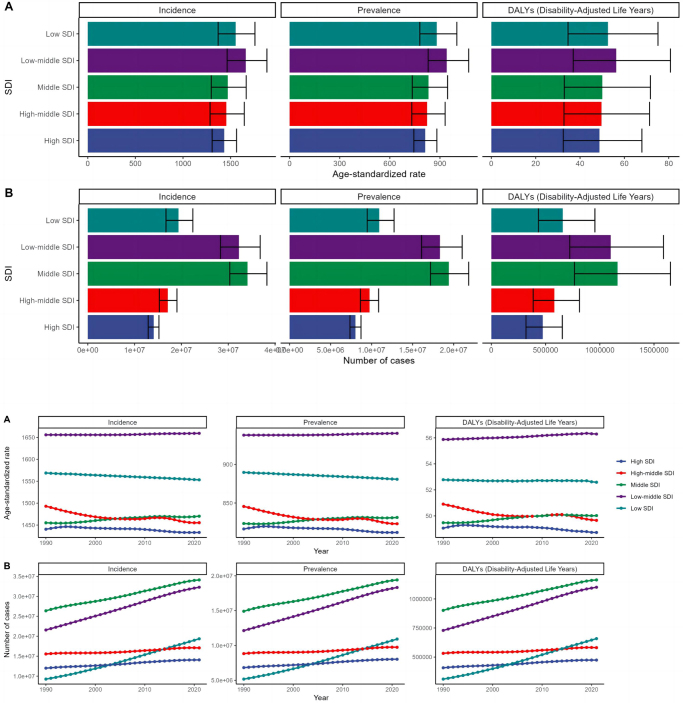
2021 GBD by Socio-Demographic Index. (A) 2021 SDI and (B) 1990–2021 SDI

In 2021, urticaria incidence varied significantly across regions with different SDI levels. The highest incidence was observed in low-middle SDI regions, with 1655.91 cases per 100 000 people (95% UI: 1460.85–1877.51), followed by low SDI regions. In contrast, high SDI regions had the lowest incidence, at 1433.17 cases per 100 000 people (95% UI: 1306.16–1562.71) (Fig. 3a, Supplementary Digital Content Table 1, available at: http://links.lww.com/JS9/E869).

In terms of prevalence, low-middle SDI regions again had the highest rate, at 938.01 cases per 100 000 people (95% UI: 826.49–1070.79), while high SDI regions had the lowest prevalence, only at 816.36 cases per 100 000 people (95% UI: 741.79–894.85) (Fig. 3b, Supplementary Digital Content Table 2, available at: http://links.lww.com/JS9/E870). Additionally, there were significant differences in DALYs associated with urticaria across SDI regions. Low SDI regions had the highest DALYs rate, at 52.59 per 100 000 people (95% UI: 34.58–75.14), resulting in a total of 658 545 DALYs (95% UI: 433 933–954 399). Low-middle SDI regions had a DALYs rate of 50.02 per 100 000 people (95% UI: 32.9–71.79), with a total of 1 100 552 DALYs (95% UI: 721 991–1 596 913). In contrast, high SDI regions had the lowest DALYs rate, at 48.72 per 100 000 people (95% UI: 32.43–67.89), resulting in 471 408 DALYs (95% UI: 319 808–654 609) (Fig. 3a, Supplementary Digital Content Table 3, available at: http://links.lww.com/JS9/E871).

In terms of the public health implications, lower-SDI regions face greater disease burden despite possibly having fewer diagnostic and treatment resources. This emphasizes the need for targeted interventions that aim to reduce the burden in low-resource settings.

### Country-level difference analysis

**Figure 4.**
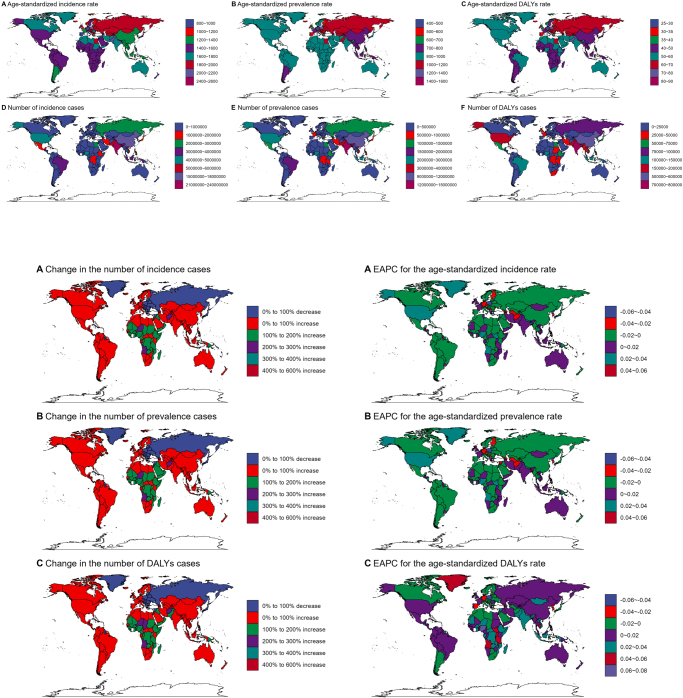
2021 GBD: country-specific age-standardized incidence, prevalence, DALYs, and 1990–2021 changes in case numbers and rates. (A) 2021 country, (B) 1990–2021 country number, and (C) 1990–2021 country rate.

At the national level, significant differences in urticaria burden were observed across countries. In 2021, Nepal had the highest global age-standardized incidence, prevalence, and DALYs rates, with an incidence rate of 2563.35 cases per 100 000 people (95% UI: 2274.38–2794.79). The high burden in Nepal and similar countries may be linked to underdeveloped healthcare systems, lack of access to treatments, and environmental factors (Fig. 4a). On the other end of the spectrum, Portugal had the lowest incidence rate of 833.63 cases per 100 000 people (95% UI: 737.02–929.89). Countries with better healthcare infrastructure, more advanced treatment protocols, and heightened public health awareness tend to have lower urticaria burdens. These findings suggest that countries with lower burdens can serve as models for those with higher burdens to improve healthcare delivery and management of urticaria.

Globally, the EAPC in incidence, prevalence, and the number of cases varies significantly across countries (Fig. 4b, c). For instance, San Marino and Andorra showed the largest upward trends in EAPC for incidence and prevalence, while Qatar and the Democratic People’s Republic of Korea exhibited the most significant downward trends.

Furthermore, there are substantial differences in DALYs between countries. For example, Nepal had the highest DALYs rate, whereas Portugal had the lowest. This reflects the considerable disparities in healthcare resource allocation, disease management, and socioeconomic development across nations (Fig. 4b, c).

### Age and gender differences

**Figure 5.**
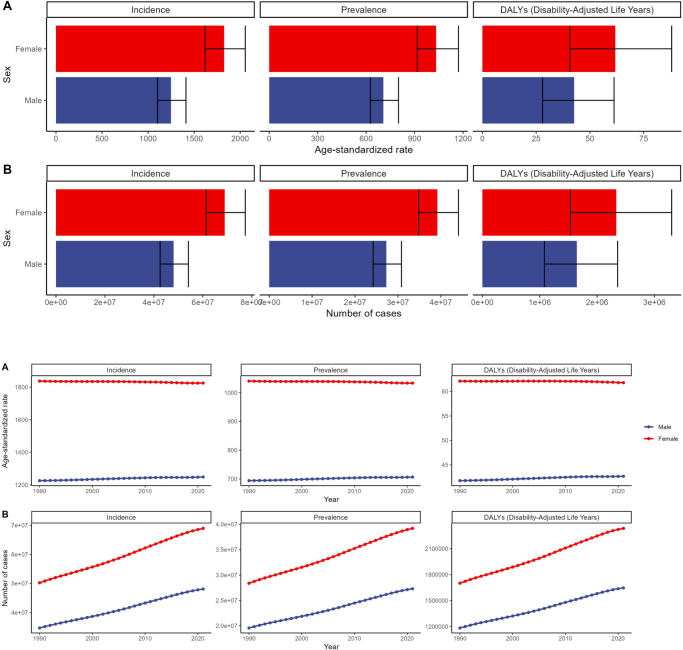
2021 GBD: sex-specific incidence, prevalence, DALYs, and changes from 1990 to 2021. (A) 2021 sex and (B) 1990–2021 sex.

Globally, the incidence, prevalence, and DALYs of urticaria show significant differences across age groups and genders. In 2021, the incidence and prevalence of urticaria were higher in females than in males, with an incidence rate of 1825.42 per 100 000 (95% UI: 1618.02–2054.93) in females, compared to 1248.68 per 100 000 (95% UI: 1102.97–1398.97) in males (Fig. 5a, b, Supplementary Digital Content Table 1, available at: http://links.lww.com/JS9/E869). This finding aligns with existing literature indicating that hormonal and immunological factors may make females more prone to urticaria.

**Figure 6.**
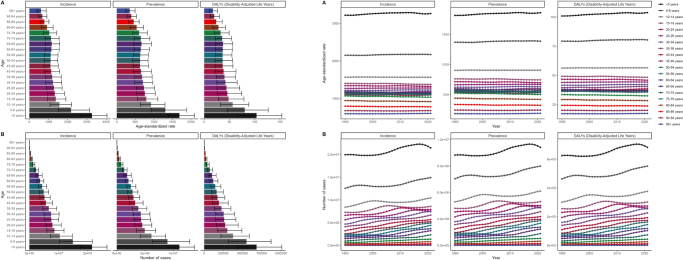
2021 GBD: age-specific incidence, prevalence, DALYs, and 1990–2021 changes across different age groups. (A) 2021 age and (B) 1990–2021 age

In terms of age, children under 5 years old had the highest incidence rate of 3262.05 cases per 100 000 people (95% UI: 2644.11–4044.43). The incidence gradually declines as age increases, contradicting earlier assumptions that an aging population would result in a higher urticaria burden. Instead, the burden is significantly lower in older populations, likely due to a decrease in exposure to allergens or improved immune regulation with age (Fig. 6).

### Predictive analysis

**Figure 7.**
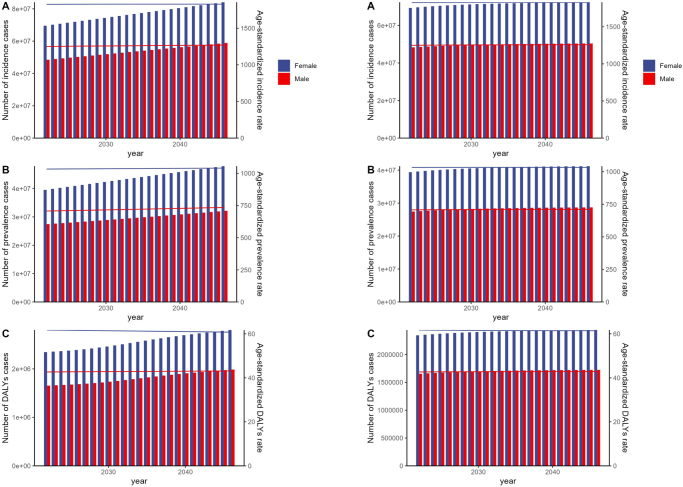
ARIMA and ES models predicting incidence, prevalence, and DALYs from 2021 to 2050. (A) ARMIA model and (B) ES model.

ARIMA model projections indicate substantial increases through 2050. For males, age-standardized incidence rates are projected to increase by 1.8% from 1250.06 to 1272.86 cases per 100 000 by 2050, while absolute incident cases will rise by 25.0% from 48.50 to 60.61 million. Male prevalence rates are projected to increase by 4.6% from 707.37 to 739.70 cases per 100 000, with prevalent cases growing by 20.1% from 27.47 to 33.00 million. Male DALYs are anticipated to increase by 23.6% from 1.65 to 2.04 million years (Fig. 7a, Supplementary Digital Content Table 4, available at: http://links.lww.com/JS9/E872).

For females, projections indicate a 0.1% increase in age-standardized incidence rates from 1825.87 to 1828.31 cases per 100 000 by 2050, while absolute incident cases will rise by 24.3% from 69.56 to 86.48 million. Female prevalence rates are expected to grow by 0.8% from 1033.62 to 1042.33 cases per 100 000, with prevalent cases increasing by 24.7% from 39.54 to 49.30 million. Female DALYs are projected to increase by 22.6% from 2.34 to 2.87 million years (Fig. 7a, Supplementary Digital Content Table 4, available at: http://links.lww.com/JS9/E872).

The ES model forecasts a similar trend, with minor increases in incidence and prevalence. This modest upward trend underscores the importance of continued monitoring and preventive interventions, especially in low-SDI regions where the projected burden is likely to be highest. Public health strategies focusing on environmental management, health education, and access to care will be critical in mitigating the future burden of urticaria (Fig. 7b, Supplementary Digital Content Table 5, available at: http://links.lww.com/JS9/E873).

Combining the two model projections suggests that the global incidence, prevalence and DALYs of urticaria are likely to show a small increase in the coming decades. Only a small acceleration of this trend may be related to various factors, such as future environmental changes, lifestyle shifts, and population aging. But its upward trend suggests that the world still needs more attention and proactive measures in public health (Supplementary Digital Content Table 6, available at: http://links.lww.com/JS9/E874).

## Discussion

Our study provides a comprehensive overview of the global burden of urticaria over the past three decades, revealing both overall stability in age-standardized rates and significant heterogeneity across demographic and geographic groups. While the global age-standardized incidence and prevalence rates have remained nearly stable (EAPC ≈ 0.01%), the absolute number of cases has increased substantially due to population growth. This disconnect between stable rates and rising case counts suggests that urticaria remains a persistent, under-addressed burden rather than an emerging epidemic, requiring sustained rather than crisis-driven responses.

Crucially, our analysis highlights pronounced regional disparities. For instance, Central and Eastern Europe consistently show the highest age-standardized rates, while Oceania and Western Europe remain comparatively low. These discrepancies may stem from a combination of genetic predisposition, differential access to dermatological care, variation in environmental allergen exposure, and differences in reporting or diagnosis. The sharp increase in South Asia, contrasted with the decline in high-income Asia Pacific, warrants deeper exploration—potential factors may include rising urban pollution, changes in healthcare infrastructure, or differences in awareness and treatment-seeking behaviors. Such divergent trends suggest that urticaria burden is sensitive to both developmental and environmental transitions.

The observed differences by SDI level underscore the social determinants of allergic disease burden. Low- and low-middle SDI countries not only bear the highest burden but may also suffer from diagnostic underreporting due to lack of access to specialists, limited public awareness, and constrained treatment options. Consequently, the true burden may be underestimated in these regions. This highlights the importance of integrating urticaria management into broader primary care and public health infrastructure, particularly in resource-limited settings^[17]^.

In terms of gender, women consistently showed higher incidence and prevalence rates than men, which aligns with previous studies suggesting that hormonal, genetic, and immunological factors may make females more susceptible to urticaria. Future research could focus on exploring these biological mechanisms to develop more targeted prevention and treatment strategies for women^[18]^.

Interestingly, children under five years old showed the highest incidence rates globally, while the burden of urticaria tends to decrease with age. This finding challenges previous assumptions that the aging population would lead to a greater urticaria burden. As the data shows, urticaria is not more common among the elderly; in fact, incidence, prevalence, and DALYs all decrease with advancing age. This highlights the importance of focusing public health efforts on younger populations, particularly children, to effectively address the highest-risk groups^[19,20]^.

Our predictive models (ARIMA and exponential smoothing) suggest that the global burden of urticaria will continue to rise modestly over the coming decades. However, the projected increases are not dramatic, with only slight changes in the incidence and prevalence rates anticipated by 2050. The small growth in urticaria burden suggests that public health efforts should focus not only on treatment but also on prevention strategies, particularly in low- and middle-SDI regions where the burden is expected to be most pronounced. Given the relatively small projected increase in disease burden, it is crucial to develop targeted interventions that address the specific needs of high-risk groups, such as children and women^[20]^. Efforts should focus on raising awareness about urticaria prevention, improving access to healthcare in lower-income regions, and optimizing resource allocation to better manage the condition.

While we want to provide a comprehensive analysis, our study has several limitations. Firstly, the analysis relies on data from the GBD 2021 database, which may vary in quality across regions. Differences in diagnostic criteria, reporting practices, and healthcare infrastructure between countries could affect the accuracy of the data^[21]^. A critical limitation of our analysis is the inability to distinguish between acute and chronic urticaria within the GBD database. This distinction is clinically and epidemiologically important, as acute urticaria (duration <6 weeks) and chronic urticaria (duration ≥6 weeks) represent distinct disease entities with different pathophysiological mechanisms, treatment requirements, and health impacts. Acute urticaria typically resolves spontaneously, while chronic urticaria requires long-term management and significantly impacts quality of life. Studies suggest chronic urticaria affects approximately 0.5–1% of the global population, while acute urticaria may affect up to 20% at some point. Our combined burden estimates likely reflect predominantly acute cases in incidence figures, while prevalence may more closely approximate chronic patterns due to persistent disease nature. Future studies should aim to address these distinctions to provide more granular insights into the global burden of urticaria. Furthermore, while environmental and lifestyle changes are hypothesized to contribute to urticaria’s increasing burden, there is no direct evidence from this study linking climate change to the rise in urticaria incidence. Therefore, any study on the effect of climate on urticaria should be considered speculative until future data are updated.

Future research should focus on understanding the regional differences in urticaria burden, distinguishing between acute and chronic urticaria, and exploring the biological mechanisms that may explain gender-based differences in susceptibility. By addressing these gaps, we can develop more effective strategies to reduce the global burden of this condition.

Overall, this study provides a systematic analysis of the current global burden of urticaria and suggests specific responses by region, gender, and age. It also projects an increase in the prevalence of urticaria from 2030 to 2050, highlighting the need for proactive measures. Future public health policies should focus on environmental improvement, resource optimization, and personalized health management, while encouraging international cooperation to address the anticipated rise in the global burden of urticaria. These measures will help mitigate the global health impact of urticaria and provide patients with a better quality of life and health protection. By preparing for the predicted increase in urticaria cases, we can ensure that the necessary healthcare infrastructure and support systems are in place to manage this growing challenge.

## Data sharing statement

GBD study 2021 data resources were available online from the Global Health Data Exchange (GHDx) query tool (http://ghdx.healthdata.org/gbd-results-tool).
